# Knowledge, Practices, and Perceived Barriers Regarding Cancer Pain Management Among Physicians and Nurses In Korea: A Nationwide Multicenter Survey

**DOI:** 10.1371/journal.pone.0105900

**Published:** 2014-08-21

**Authors:** Hyun Jung Jho, Yeol Kim, Kyung Ae Kong, Dae Hyun Kim, Jin Young Choi, Eun Jeong Nam, Jin Young Choi, Sujin Koh, Kwan Ok Hwang, Sun Kyung Baek, Eun Jung Park

**Affiliations:** 1 Hospice & Palliative Care Branch, National Cancer Control Institute, National Cancer Center, Goyang, Republic of Korea; 2 Division of Cancer Management & Policy, National Cancer Control Institute, National Cancer Center, Goyang, Republic of Korea; 3 Clinical Trial Center, Ewha Womans University Medical Center, Seoul, Republic of Korea; 4 Department of Pain and Anesthesiology, Hospital, National Cancer Center, Goyang, Republic of Korea; 5 Department of Nursing, Hospital, National Cancer Center, Goyang, Republic of Korea; 6 Department of Hematology and Oncology, Ulsan University Hospital, Ulsan, Republic of Korea; 7 Korean Hospice & Palliative Nurse Association, Daejeon, Republic of Korea; 8 Department of Medical Oncology and Hematology, Kyung Hee University Medical Center, Seoul, Republic of Korea; University of Michigan, United States of America

## Abstract

**Purpose:**

Medical professionals’ practices and knowledge regarding cancer pain management have often been cited as inadequate. This study aimed to evaluate knowledge, practices and perceived barriers regarding cancer pain management among physicians and nurses in Korea.

**Methods:**

A nationwide questionnaire survey was administered to physicians and nurses involved in the care of cancer patients. Questionnaire items covered pain assessment and documentation practices, knowledge regarding cancer pain management, the perceived barriers to cancer pain control, and processes perceived as the major causes of delay in opioid administration.

**Results:**

A total of 333 questionnaires (149 physicians and 284 nurses) were analyzed. Nurses performed pain assessment and documentation more regularly than physicians did. Although physicians had better knowledge of pain management than did nurses, both groups lacked knowledge regarding the side effects and pharmacology of opioids. Physicians working in the palliative care ward and nurses who had received pain management education obtained higher scores on knowledge. Physicians perceived patients’ reluctance to take opioids as a barrier to pain control, more so than did nurses, while nurses perceived patients’ tendency to under-report of pain as a barrier, more so than did physicians. Physicians and nurses held different perceptions regarding major cause of delay during opioid administration.

**Conclusions:**

There were differences between physicians and nurses in knowledge and practices for cancer pain management. An effective educational strategy for cancer pain management is needed in order to improve medical professionals’ knowledge and clinical practices.

## Introduction

Pain management is an important aspect in the care of cancer patients. Although cancer pain can be controlled up to 90% of the cases through the use of appropriate methods [1],[2], its prevalence has been reported to be as high as 64% among patients with advanced cancer [3] and pain control was found to be inadequate for more than 40% of patients [4]. Factors relating to the medical professional, patient, and the healthcare system have been identified as causes of this apparent under-treatment of cancer pain among patients [4]. Specifically, medical professionals’ inadequacy in pain assessment and management has been pointed out as an important barrier to cancer pain control [5]–[9].

Studies have noted knowledge deficits in cancer pain management among medical professionals [10],[11]. For example, physicians, especially oncologists, generally have general knowledge of cancer pain management, while seemingly lacking knowledge regarding opioid administration or alternate therapies for pain control [8]. This knowledge deficit typically emerged when physicians calculated opioid dosages for the management of breakthrough pain [12] and when attempting to select the correct response to challenging clinical vignettes [5]. Nurses’ knowledge of opioids as a pain control measure was found to be as inadequate as that of the physicians referred to above [8], demonstrated by their exaggerated fear of the addictive nature of opioids or their potential to culminate in respiratory suppression [6]. In Korea, studies also have reported inadequate knowledge and incorrect attitudes among doctors and nurses [13]–[16]. Kim et al. surveyed 1,204 young doctors from all specialties and determined that the majority of doctors were not knowledgeable about equianalgesic opioid dosages, adjuvant analgesics, the numeric rating scale, and the fact that opioids have no ceiling effect [13].

In Korea, approximately 200,000 people are diagnosed with cancer annually, while more than 70,000 die from the condition [17]. According to another Korean study, 70% of patients with advanced cancer experienced pain, and pain management was inadequate in half of the patients [18].

Cancer pain control forms part of Korea’s national healthcare policy. In order to improve knowledge and practices regarding cancer pain management among medical professionals, Korea’s Ministry of Health and Welfare and the National Cancer Center have been publishing and distributing a national guideline known as the “Cancer Pain Management Guideline” across Korea since 2004. In the current study, we investigated and compared knowledge, practices and perceived barriers regarding cancer pain management among physicians and nurses to identify the need for improvement in clinical practice, education and policy.

## Method

### Study Design, Participants, And Procedures

This survey took place from September 2010 until June 2011 at 11 hospitals (6 public and 5 private hospitals) across Korea. Doctors and nurses involved in the care of cancer patients were eligible for participation. The research coordinator contacted eligible participants at participating hospitals and subsequently delivered the questionnaires. All participants provided written consent to participate in the study. Upon completion of the questionnaire, participants received a voucher worth 10,000 KRW (approximately US $10) as a reward for their participation. This study was approved by the Institutional Review Board at 11 participating hospitals (National Cancer Center, Chonbuk National University Hospital, Chonnam National University Hospital, Chungbuk National University Hospital, Chungnam National University Hospital, Pusan National University Hospital, Good Samaritan Hospital, Korea Cancer Center Hospital, Korea University Kuro hospital, KyungHee University Medical Center, The Catholic University of Korea Seoul St. Mary’s Hospital).

### Questionnaire Development

The questionnaire was developed by the researchers for the purposes of the current study and pilot-tested and revised by a panel of experts consist of 7 physicians (from specialty of pain, medical oncology, family medicine and preventive medicine) and 8 nurses working in oncology ward or palliative care unit. Questionnaire items relating to knowledge and practices regarding cancer pain management were generated on the basis of the contents of the Cancer Pain Management Guideline published by the Ministry of Health and Welfare and the National Cancer Center. The Guideline encompasses pain assessment, pharmacologic and non-pharmacologic pain management, radiation therapy for pain control, interventional pain management such as nerve block, and approaches towards refractory cancer pain.

Items relating to practices assessed the frequency of pain assessment for admitted patients, specific details of pain assessment, and documentation following assessment. Knowledge of cancer pain management was evaluated through 14 items (11 “true” or “false” questions and 3 multiple choice questions), tapping into the principles of cancer pain management, specific properties of analgesics, interventional pain management, opioid dose calculation, and the duration of re-assessment after opioid administration.

Questions on perceived barriers were extracted from the literature [19]–[23], and comprised 3 categories related to patients, medical staff, and the health care system. The following is an example of an item relating to perceived barriers: “how often does a particular barrier interfere with pain control?” Responses were organized on a 4-point scale, where a scale of 1 indicated “never,” 2 = “sometimes,” 3 = “often,” and 4 = “always.” For each item, frequency of scale 1 and 2 was combined as negative response, and frequency of scale 3 and 4 was combined as positive response for a statistical analysis.

### Data Analysis

Descriptive analysis was used to summarize the characteristics of participants and responses to items relating practice, knowledge, and perceived barriers. A chi-squared test was carried out to compare differences between physicians and nurses in terms of practices, knowledge, and perceived barriers to cancer pain control. We used multiple linear regression analysis to determine the relationship between participants’ characteristics and the number of correct responses for knowledge of cancer pain management. β value is a regression coefficient which is interpreted as the difference in the number of correct answers according to the differences in age, sex, whether the participant is working in palliative care unit, and whether the participant had attended any type of cancer pain education. The statistical significance level was set at p<0.05. Statistical analyses were performed using the STATA SE version 12.0 software package (StataCorp, College Station, TX, USA).

## Results

### Sample Characteristics

A summary of the study participants’ characteristics is presented in Table 1. A total of 335 questionnaires were collected from 11 hospitals. After excluding 2 incomplete questionnaires, 333 (149 physicians and 284 nurses) questionnaires were included in the final analysis. The participants’ mean age was 33.2 and 29.0 (in years) for physicians and nurses, respectively. 70.8% of participating physicians specialized in internal medicine. 13.3% of physicians and 19.0% of nurses were working in the palliative care ward. More than 70% of physicians and nurses had received cancer pain education.

**Table 1:** Characteristics of participants.

	Physician, N = 149	Nurse, N = 284
Characteristics	n (%)	n (%)
Gender		
Male	91 (61.5)	0 (0)
Female	57 (38.5)	283 (100)
Age (years) Mean (SD)	33.2 (6.9)	29.0 (5.8)
<40	119 (85.6)	251 (92.9)
≥40	20 (14.4)	19 (7.0)
Working experiencein years, Mean (SD)	7.3 (6.5)	6.6 (5.8)
<10	111 (75.5)	207 (76.7)
≥10	36 (24.5)	63 (23.3)
Clinical specialty		
Internal medicine	104 (70.8)	
Surgery	24 (16.3)	
Family medicine	9 (6.1)	
Other*	10 (6.8)	
Working in palliative care unit	20 (13.3)	54 (19.0)
Having attended any type ofcancer pain education	106 (72.1)	232 (78.8)

*Other includes gynecology, radiation oncology, and odontology.

### Pain Assessment Practices And Documentation

Table 2 presents practices relating to pain assessment and documentation among physicians and nurses. Results showed that about three times more nurses indicated that they assessed patients’ pain levels on every round than physicians did (p<0.001). In addition, nurses checked all items relating to pain assessment more frequently than physicians did. Further, a higher proportion of nurses, in comparison to physicians, reported that they documented pain assessment (p<0.001). In additional analysis, there was no significant difference in general characteristics among nurses according to the pain assessment and documentation practices. For physicians, having received cancer pain education and working in the palliative ward were positively associated with doing pain documentation (p = 0.001).

**Table 2:** Pain assessment and documentation practices.

	Physician	Nurse	P value
	%	%	
Occasion of painassessment			
Every round*	24.8	75.2	<0.001
On selected occasions* †	67.1	23.2	<0.001
Seldom*	4.0	0.4	0.015
Items checked duringpain assessment			
Location*	83.8	95.8	<0.001
Quality*	66.9	89.8	<0.001
Related factor*	55.4	68.8	0.006
Severity*	84.4	97.2	<0.001
Timing*	69.4	78.8	0.031
Documentation ofpain assessment*	60.8	98.9	<0.001

*P<0.05.

† New admission, when patient complains of pain, or when patient seems to be in pain.

### Knowledge Of Cancer Pain Management

The rate of correct responses to each of the questions on knowledge for cancer pain management is shown in Table 3. The mean number of correct responses provided by physicians was higher than that of nurses, at 10.8 and 9.0, respectively. For both physicians and nurses, knowledge deficit was prominent in questions on tolerance for opioid-induced sedation (Question 8: the rate of correct responses was 48.0% and 35.0% for physicians and nurses, respectively) and the duration of re-assessment after intravenous morphine administration (Question 13: 51.0% and 48.4% for physicians and nurses, respectively). The rate of correct responses was higher among physicians for questions on NSAIDs (Question 3), specific properties of opioids and opioid dose calculation (Questions 7, 10, and 12), as well as the utilization of radiotherapy or interventional pain management as measures of pain control (Questions 9 and 11).

**Table 3:** Rate of correct responses regarding knowledge of cancer pain management.

	Physician	Nurse	P value
Question	%	%	
1. You should not trust patient’ssubjective reports of pain (F)	86.5	91.3	0.118
2. You should differentiable certain causeof pain which needs specific treatment(i.e. cord compression) (T)	96.6	96.7	0.943
3. Prescribing a few differenttypes of NSAIDs will increasethe analgesic efficacyand decreased adverse effect* (F)	77.7	52.7	<0.001
4. Pethidine can be prescribedfor chronic cancer pain safely. (F)	81.8	81.2	0.880
5. Opioid analgesics have ahigh risk of addiction. (F)	91.9	86.3	0.087
6. The effect of immediate releaseoral opioid can be assessed at1 hour after administration (T)	77.0	76.8	0.960
7. Opioid analgesics do not have aceiling effect (T)*	78.4	55.2	<0.001
8. Tolerance for opioid-induced sedationdevelops within a few days* (T)	48.0	35.0	0.008
9. For painful bone metastasis,radiotherapy can alleviate the pain or helpto reduce the amount of analgesics* (T)	82.3	53.1	<0.001
10. Opioid-induced respiratorysuppression is common* (F)	82.4	54.9	<0.001
11. Celiac plexus block is effective fortreating cancer pain at upper abdomen.* (T)	68.5	45.3	<0.001
12. Calculation of opioid rescue dose*	73.1	52.4	<0.001
13. Duration of evaluation followingintravenous morphine administration	51.0	48.4	0.607
14. Knowledge regardingrefractory cancer pain	96.0	93.6	0.314
Mean number of correctresponses*, Mean (SD)	10.8 (2.4)	9.0 (2.5)	<0.001

*P<0.05.

A relationship between the number of correct responses given on knowledge of cancer pain management and the sample characteristics is shown in Table 4. Working in palliative care unit was associated with a higher number of correct responses for physicians. For nurses, being older and having received cancer pain education were related to a higher rate of correct responses.

**Table 4:** Relationship between knowledge and characteristics of participants.

	Physician	Nurse
	β (95% CI)	β (95% CI)
Age	NS	0.07 (0.02, 0.12)
Sex*	NS	-
Working in palliative care unit^†^	1.25 (0.05, 2.44)	NS
Having attended any type of cancer pain education^‡^	NS	1.75 (0.95, 2.56)

Multiple linear regression analysis for the number of correct answers for knowledge with general characteristics of participants as independent variables.

β regression coefficient; NS not significant.

Reference values: *Male; ^†^Not working in the palliative care ward; ^‡^Not having attended any type of cancer pain education.

### Perceived Barriers For Cancer Pain Management

Perceived barriers for cancer pain control were compared; results are described in Table 5. Time constraints and insufficient knowledge of pain control were cited by both nurses and physicians as barriers to cancer pain management that were experienced more frequently than other barriers. Physicians perceived barriers such as the “patient’s reluctance to take opioids” (p = 0.011) and “not prioritizing pain control” (p = 0.003) to a higher extent than nurses. In contrast, nurses perceived “insufficient communication with patients” (p = 0.001) and “patients’ reluctance to report pain” (p = 0.030) as barriers to a higher extent than physicians.

**Table 5:** Perceived barriers to cancer pain control.

	Physician	Nurse	P value
	%	%	
Related to medical staff			
Inadequate pain assessment	37.6	36.8	0.864
Inadequate experienceon pain control	42.3	35.7	0.180
Insufficient knowledgeof pain control	45.0	40.8	0.403
Time constraints*	75.8	66.1	0.036
Reluctance to prescribeopioid	23.5	25.8	0.599
Insufficient communicationwith patient*	30.2	47.0	0.001
Patient-related			
Reluctance to report pain*	36.1	47.0	0.030
Reluctance to take opioid*	54.1	41.1	0.011
Insufficient communicationwith medical staff	44.6	49.1	0.373
Financial constraints	12.8	13.3	0.891
Insufficient knowledge ofpain control	60.5	57.7	0.572
Related to the health care system			
Strict regulation of opioids	29.1	36.5	0.120
Inadequate staffing	41.9	39.7	0.662
Limited stock of differenttypes of opioids	29.1	27.1	0.675
Cancer pain management is notconsidered as important*	43.5	29.3	0.003
Medication and intervention costs*	32.7	23.6	0.044

*P<0.05.

### Perceptions Regarding Processes Delaying Opioid Administration

**Figure 1:**
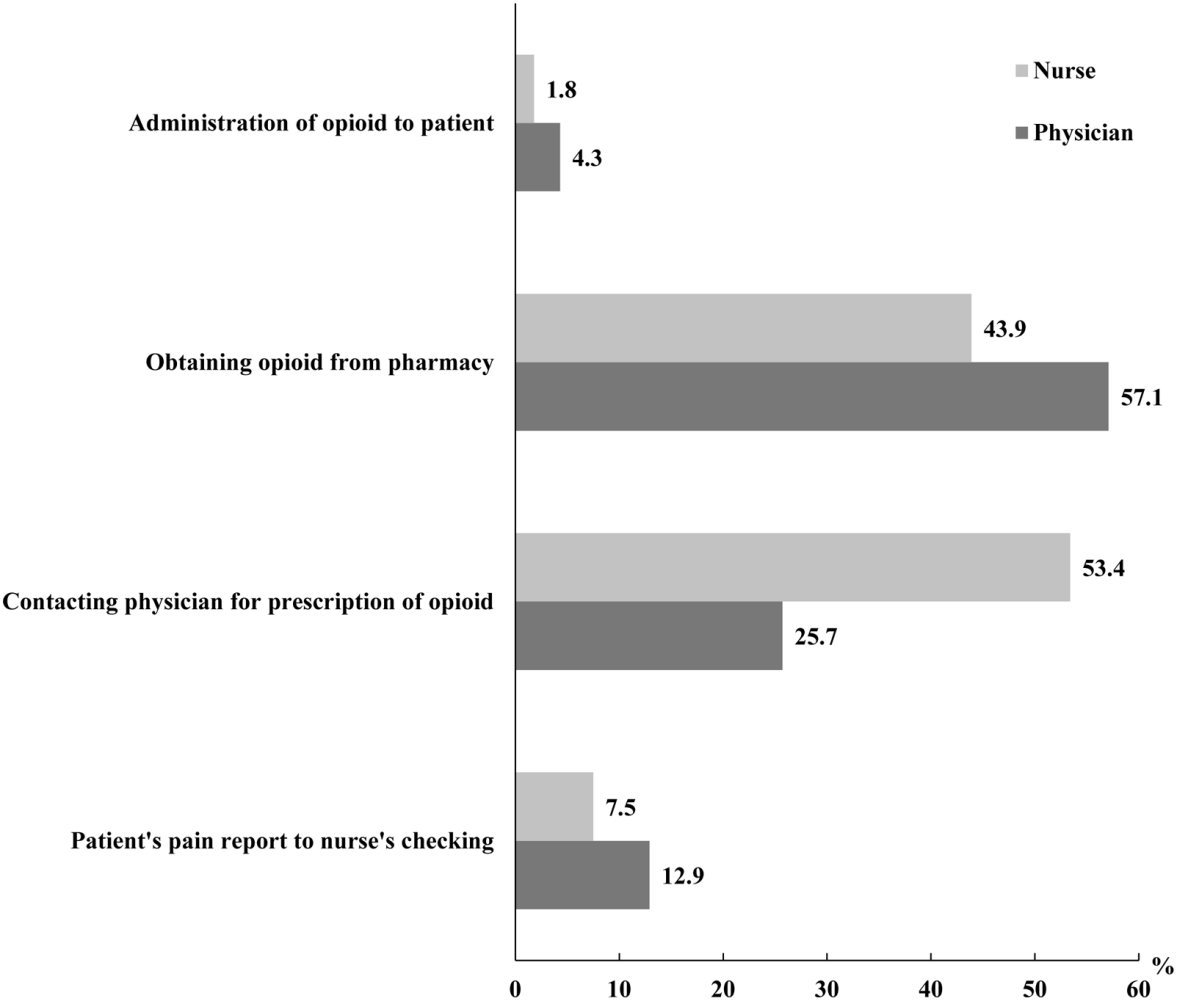
The most delayed process during opioid administration perceived by physicians and nurses.

Figure 1 depicts differences in perceptions regarding processes considered to be major sources of delay in the administration of opioids between physicians and nurses. In this regard, “obtaining opioids from pharmacy” was cited by 57.1% of physicians, while 53.4% of nurses cited “contacting physician for prescription of opioid” (p<0.001).

## Discussion

This is the first nationwide study to evaluate knowledge and practices relating to cancer pain management among doctors and nurses in Korea, on the basis of the national Cancer Pain Management Guideline. Overall, results showed that doctors have better knowledge of cancer pain management than nurses, while the latter tended to conduct regular pain assessment and documentation practice more frequently. In addition, doctors and nurses held different perceptions regarding barriers to cancer pain management and the opioid administration process.

The differences found between nurses and doctors in knowledge and practices reflect areas requiring improvement in cancer pain management within these professions. Similarly to results from previous studies [10],[24],[25], our findings showed that doctors scored higher on knowledge than nurses. In our study, both doctors and nurses displayed substantial knowledge deficits regarding tolerance for opioid-induced sedation, the duration of evaluation after opioid administration, calculation of rescue opioid dosages, and utilization of other modalities, such as celiac plexus block. This result is also consistent with findings from previous studies [5],[8],[12]. The low rate of correct responses obtained for knowledge regarding celiac plexus block indicates limited understanding of specialist areas within the medical field, which could lead to less referrals to other specialists. It was reported in a previous study that less than 20% of oncologists referred their patients to palliative or pain specialists [5]. Moreover, medical staff with insufficient knowledge of pain control may delay the administration of opioids to patients [15].

It is generally agreed that the education of medical professionals is necessary to improve cancer pain management. Findings from our study showed that having received cancer pain education was significantly associated with better knowledge among nurses, which was compatible with findings from other study [26]. However, this benefit of education is unclear among physicians. In our finding, knowledge regarding cancer pain management was not significantly different between physicians who received cancer pain education and who didn’t. This echoes with previous study [5] which showed increased continued medical education for pain management could not satisfy the clinical need in pain management. In comparison, projects based approaches which utilized developing action plan [27] or continued quality improvement [28] for pain management reported improved practice and knowledge among medical professionals. This suggests that delivering knowledge alone is not enough to promote cancer pain management, and additional educational strategy such as combining different education methods is needed [29]. For example, the didactic lecture is used for delivering general principles, while small group discussions are more suitable for discussions of clinical cases [29]. This issue relating to educational methods seems to be applicable to the Korean context, where didactic lectures are most widely used for medical education.

In the current study, nurses practiced pain assessment and documentation more regularly than physicians. This is compatible with findings from a previous study which reported that nurses were more skilled at pain assessment, as compared to doctors or pharmacists [8]. This difference could be explained by the roll allocation between nurses and physicians, since nurses are usually required to monitor and report patient’s symptom to physicians. Another consideration which could have influenced nurses’ pain assessment and documentation practice in our survey is the institutional accreditation standard. In this regard, all but one participating hospitals in the current study were accredited by Korea Institute for Healthcare Accreditation (KOIHA), which recommends to assess pain using assessment tool for inpatients.

Time constraints and insufficient knowledge regarding pain management of medical professional were the most commonly encountered barriers to effective pain management for both physicians and nurses. Interestingly, physicians and nurses held different perceptions regarding patient-related barriers. Physicians perceived the “patient’s reluctance to take opioid” as a barrier more often, while nurses perceived the “patient underreporting pain” more often. This is comparable to findings from a previous study, wherein doctors tended to believe that patients over-reported their pain, more so than nurses [8].

There were differences between the nurses and the physicians in perceptions regarding factors that contributed the most towards the delay in the opioid administration process. This discordance of perception between physicians and nurses seems to reflect insufficient coordination of the opioid administration process. For example, in many hospital wards in Korea, nurses are often required to confirm the administration of opioids with physicians, even when there is an “as needed” opioid prescription for breakthrough pain. Moreover, opioids are usually kept in hospital pharmacies, rather than wards, due to the strict regulation policy. In such instances, traveling between the pharmacy and the ward to obtain opioids is time-consuming. In order to reduce the duration of this process, there is a need for institutional policy change, which could advocate the storage of opioids in hospital wards as well as clear team communication about executing standing order for breakthrough pain.

This study has a few limitations. First, although we performed a multicenter study, the results may not represent practices and knowledge of all medical professionals in Korea. Second, in order to accurately measure actual pain assessment and documentation practices, additional monitoring, such as auditing charts, is needed. Third, in this study, we simply asked whether participants had attended pain education before, so as to determine exposure to the educational experience, without considering the level of such exposure. However, the content and methods used in cancer pain management education could be diverse; this, however, was not considered in the current study. Finally, the actual duration of the each process for the administration of opioid was not measured. Therefore we couldn’t verify the medical professional’s perception for the delayed process.

In conclusion, physicians showed better knowledge of cancer pain management, while nurses performed better in pain assessment and documentation practices. Although time constraints were perceived more frequently as a barrier to cancer pain management, physicians perceived patients’ reluctance to take opioids as a barrier, more so than nurses, while nurses deemed patients’ underreporting of pain as a barrier to pain control, more so than physicians. The findings in this study indicate that changes in the educational strategy are required to enhance clinical practice among healthcare professionals in relation to cancer pain management.
